# Supplementary material for: Resolving the full spectrum of human genome variation using Linked-Reads
Source: Genome Res. 2019 Apr;29(4):635–45. doi: 10.1101/gr.234443.118 (PMC6442396; doi:10.1101/gr.234443.118)
Supplement: Supplemental Material [file supp_gr.234443.118_Supplemental_longranger-2.2.zip › longranger-2.2.tar.bz2!/lr2.2-public/lib/stan/cmdstan/stan/doc/api/html/base__nuts_8hpp_source.html]

Stan: src/stan/mcmc/hmc/nuts/base\_nuts.hpp Source File


|  |  |
| --- | --- |
|  | Stan  2.5.0  probability, sampling & optimization |


- Main Page
- Related Pages
- Namespaces
- Classes
- Files

- File List
- File Members

All Classes Namespaces Files Functions Variables Typedefs Enumerations Enumerator Friends Macros Pages

- src
- stan
- mcmc
- hmc
- nuts

base\_nuts.hpp

Go to the documentation of this file.

1 #ifndef STAN\_\_MCMC\_\_BASE\_\_NUTS\_\_BETA

2 #define STAN\_\_MCMC\_\_BASE\_\_NUTS\_\_BETA

3

4 #include <math.h>

5 #include <boost/math/special\_functions/fpclassify.hpp>

6 #include <stan/math/functions/min.hpp>

7 #include <stan/mcmc/hmc/base\_hmc.hpp>

8 #include <stan/mcmc/hmc/hamiltonians/ps\_point.hpp>

9

10 namespace stan {

11

12  namespace mcmc {

13

14  struct nuts\_util

15  {

16  // Constants through each recursion

17  double log\_u;

18  double H0;

19  int sign;

20

21  // Aggregators through each recursion

22  int n\_tree;

23  double sum\_prob;

24  bool criterion;

25

26  };

27

28  // The No-U-Turn Sampler (NUTS).

29

30  template <class M, class P, template<class, class> class H,

31  template<class, class> class I, class BaseRNG>

32  class base\_nuts: public base\_hmc<M, P, H, I, BaseRNG>

33  {

34

35  public:

36

37  base\_nuts(M &m, BaseRNG& rng, std::ostream\* o, std::ostream\* e):

38  base\_hmc<M, P, H, I, BaseRNG>(m, rng, o, e),

39  depth\_(0), max\_depth\_(5), max\_delta\_(1000), n\_leapfrog\_(0), n\_divergent\_(0)

40  {};

41

42  ~base\_nuts() {};

43

44  void set\_max\_depth(const int d) {

45  if(d > 0)

46  max\_depth\_ = d;

47  }

48

49  void set\_max\_delta(const double d) {

50  max\_delta\_ = d;

51  }

52

53  int get\_max\_depth() { return this->max\_depth\_; }

54  double get\_max\_delta() { return this->max\_delta\_; }

55

56  sample transition(sample& init\_sample) {

57

58  // Initialize the algorithm

59  this->sample\_stepsize();

60

61  nuts\_util util;

62

63  this->seed(init\_sample.cont\_params());

64

65  this->hamiltonian\_.sample\_p(this->z\_, this->rand\_int\_);

66  this->hamiltonian\_.init(this->z\_);

67

68  ps\_point z\_plus(this->z\_);

69  ps\_point z\_minus(z\_plus);

70

71  ps\_point z\_sample(z\_plus);

72  ps\_point z\_propose(z\_plus);

73

74  int n\_cont = init\_sample.cont\_params().size();

75

76  Eigen::VectorXd rho\_init = this->z\_.p;

77  Eigen::VectorXd rho\_plus(n\_cont); rho\_plus.setZero();

78  Eigen::VectorXd rho\_minus(n\_cont); rho\_minus.setZero();

79

80  util.H0 = this->hamiltonian\_.H(this->z\_);

81

82  // Sample the slice variable

83  util.log\_u = std::log(this->rand\_uniform\_());

84

85  // Build a balanced binary tree until the NUTS criterion fails

86  util.criterion = true;

87  int n\_valid = 0;

88

89  this->depth\_ = 0;

90  this->n\_divergent\_ = 0;

91

92  util.n\_tree = 0;

93  util.sum\_prob = 0;

94

95  while (util.criterion && (this->depth\_ <= this->max\_depth\_) ) {

96

97  // Randomly sample a direction in time

98  ps\_point\* z = 0;

99  Eigen::VectorXd\* rho = 0;

100

101  if (this->rand\_uniform\_() > 0.5) {

102  z = &z\_plus;

103  rho = &rho\_plus;

104  util.sign = 1;

105  }

106  else {

107  z = &z\_minus;

108  rho = &rho\_minus;

109  util.sign = -1;

110  }

111

112  // And build a new subtree in that direction

113  this->z\_.ps\_point::operator=(\*z);

114

115  int n\_valid\_subtree = build\_tree(depth\_, \*rho, 0, z\_propose, util);

116  ++(this->depth\_);

117

118  \*z = this->z\_;

119

120  // Metropolis-Hastings sample the fresh subtree

121  if (!util.criterion)

122  break;

123

124  double subtree\_prob = 0;

125

126  if (n\_valid) {

127  subtree\_prob = static\_cast<double>(n\_valid\_subtree) /

128  static\_cast<double>(n\_valid);

129  } else {

130  subtree\_prob = n\_valid\_subtree ? 1 : 0;

131  }

132

133  if (this->rand\_uniform\_() < subtree\_prob)

134  z\_sample = z\_propose;

135

136  n\_valid += n\_valid\_subtree;

137

138  // Check validity of completed tree

139  this->z\_.ps\_point::operator=(z\_plus);

140  Eigen::VectorXd delta\_rho = rho\_minus + rho\_init + rho\_plus;

141

142  util.criterion = compute\_criterion(z\_minus, this->z\_, delta\_rho);

143

144  }

145

146  this->n\_leapfrog\_ = util.n\_tree;

147

148  double accept\_prob = util.sum\_prob / static\_cast<double>(util.n\_tree);

149

150  this->z\_.ps\_point::operator=(z\_sample);

151  return sample(this->z\_.q, - this->z\_.V, accept\_prob);

152

153  }

154

155  void write\_sampler\_param\_names(std::ostream& o) {

156  o << "stepsize\_\_,treedepth\_\_,n\_leapfrog\_\_,n\_divergent\_\_,";

157  }

158

159  void write\_sampler\_params(std::ostream& o) {

160  o << this->epsilon\_ << "," << this->depth\_ << ","

161  << this->n\_leapfrog\_ << "," << this->n\_divergent\_ << ",";

162  }

163

164  void get\_sampler\_param\_names(std::vector<std::string>& names) {

165  names.push\_back("stepsize\_\_");

166  names.push\_back("treedepth\_\_");

167  names.push\_back("n\_leapfrog\_\_");

168  names.push\_back("n\_divergent\_\_");

169  }

170

171  void get\_sampler\_params(std::vector<double>& values) {

172  values.push\_back(this->epsilon\_);

173  values.push\_back(this->depth\_);

174  values.push\_back(this->n\_leapfrog\_);

175  values.push\_back(this->n\_divergent\_);

176  }

177

178  virtual bool compute\_criterion(ps\_point& start, P& finish, Eigen::VectorXd& rho) = 0;

179

180  // Returns number of valid points in the completed subtree

181  int build\_tree(int depth, Eigen::VectorXd& rho,

182  ps\_point\* z\_init\_parent, ps\_point& z\_propose,

183  nuts\_util& util)

184  {

185

186  // Base case

187  if (depth == 0)

188  {

189

190  this->integrator\_.evolve(this->z\_, this->hamiltonian\_,

191  util.sign \* this->epsilon\_);

192

193  rho += this->z\_.p;

194

195  if (z\_init\_parent) \*z\_init\_parent = this->z\_;

196  z\_propose = this->z\_;

197

198  double h = this->hamiltonian\_.H(this->z\_);

199  if (boost::math::isnan(h)) h = std::numeric\_limits<double>::infinity();

200

201  util.criterion = util.log\_u + (h - util.H0) < this->max\_delta\_;

202  if (!util.criterion) ++(this->n\_divergent\_);

203

204  util.sum\_prob += stan::math::min(1, std::exp(util.H0 - h));

205  util.n\_tree += 1;

206

207  return (util.log\_u + (h - util.H0) < 0);

208

209  }

210  // General recursion

211  else

212  {

213

214  Eigen::VectorXd left\_subtree\_rho(rho.size()); left\_subtree\_rho.setZero();

215  ps\_point z\_init(this->z\_);

216

217  int n1 = build\_tree(depth - 1, left\_subtree\_rho, &z\_init, z\_propose, util);

218

219  if (z\_init\_parent) \*z\_init\_parent = z\_init;

220

221  if (!util.criterion) return 0;

222

223  Eigen::VectorXd right\_subtree\_rho(rho.size()); right\_subtree\_rho.setZero();

224  ps\_point z\_propose\_right(z\_init);

225

226  int n2 = build\_tree(depth - 1, right\_subtree\_rho, 0, z\_propose\_right, util);

227

228  double accept\_prob = static\_cast<double>(n2) /

229  static\_cast<double>(n1 + n2);

230

231  if ( util.criterion && (this->rand\_uniform\_() < accept\_prob) )

232  z\_propose = z\_propose\_right;

233

234  Eigen::VectorXd& subtree\_rho = left\_subtree\_rho;

235  subtree\_rho += right\_subtree\_rho;

236

237  rho += subtree\_rho;

238

239  util.criterion &= compute\_criterion(z\_init, this->z\_, subtree\_rho);

240

241  return n1 + n2;

242

243  }

244

245  }

246

247  int depth\_;

248  int max\_depth\_;

249  double max\_delta\_;

250

251  int n\_leapfrog\_;

252  int n\_divergent\_;

253

254  };

255

256  } // mcmc

257

258 } // stan

259

260

261 #endif

stan::mcmc::nuts\_util

**Definition:** base\_nuts.hpp:14

boost::math::isnan

bool isnan(const stan::agrad::var &v)

Checks if the given number is NaN.

**Definition:** boost\_fpclassify.hpp:69

stan::mcmc::nuts\_util::H0

double H0

**Definition:** base\_nuts.hpp:18

stan::mcmc::base\_hmc::z\_

P z\_

**Definition:** base\_hmc.hpp:133

min.hpp

base\_hmc.hpp

stan::mcmc::base\_nuts::max\_depth\_

int max\_depth\_

**Definition:** base\_nuts.hpp:248

stan::mcmc::base\_hmc::hamiltonian\_

H< M, BaseRNG > hamiltonian\_

**Definition:** base\_hmc.hpp:135

stan::mcmc::ps\_point

**Definition:** ps\_point.hpp:16

stan::mcmc::base\_nuts::compute\_criterion

virtual bool compute\_criterion(ps\_point &start, P &finish, Eigen::VectorXd &rho)=0

stan::mcmc::base\_nuts::transition

sample transition(sample &init\_sample)

**Definition:** base\_nuts.hpp:56

stan::mcmc::base\_nuts::get\_sampler\_param\_names

void get\_sampler\_param\_names(std::vector< std::string > &names)

**Definition:** base\_nuts.hpp:164

stan::mcmc::base\_hmc::sample\_stepsize

void sample\_stepsize()

**Definition:** base\_hmc.hpp:125

stan::mcmc::base\_hmc::z

P & z()

**Definition:** base\_hmc.hpp:109

stan::mcmc::base\_nuts::write\_sampler\_param\_names

void write\_sampler\_param\_names(std::ostream &o)

**Definition:** base\_nuts.hpp:155

stan::mcmc::nuts\_util::sign

int sign

**Definition:** base\_nuts.hpp:19

stan::mcmc::base\_nuts

**Definition:** base\_nuts.hpp:32

stan::mcmc::sample::cont\_params

double cont\_params(int k) const

**Definition:** sample.hpp:30

stan::mcmc::base\_nuts::~base\_nuts

~base\_nuts()

**Definition:** base\_nuts.hpp:42

stan::mcmc::base\_nuts::get\_max\_delta

double get\_max\_delta()

**Definition:** base\_nuts.hpp:54

ps\_point.hpp

stan::mcmc::base\_hmc::rand\_int\_

BaseRNG & rand\_int\_

**Definition:** base\_hmc.hpp:137

stan::mcmc::base\_nuts::n\_divergent\_

int n\_divergent\_

**Definition:** base\_nuts.hpp:252

stan::mcmc::base\_nuts::n\_leapfrog\_

int n\_leapfrog\_

**Definition:** base\_nuts.hpp:251

stan::mcmc::base\_nuts::depth\_

int depth\_

**Definition:** base\_nuts.hpp:247

stan::mcmc::base\_hmc

**Definition:** base\_hmc.hpp:20

stan::mcmc::base\_nuts::get\_max\_depth

int get\_max\_depth()

**Definition:** base\_nuts.hpp:53

stan::mcmc::nuts\_util::criterion

bool criterion

**Definition:** base\_nuts.hpp:24

stan::mcmc::base\_nuts::base\_nuts

base\_nuts(M &m, BaseRNG &rng, std::ostream \*o, std::ostream \*e)

**Definition:** base\_nuts.hpp:37

stan::math::e

double e()

Return the base of the natural logarithm.

**Definition:** constants.hpp:86

stan::mcmc::base\_nuts::set\_max\_depth

void set\_max\_depth(const int d)

**Definition:** base\_nuts.hpp:44

stan::mcmc::base\_nuts::get\_sampler\_params

void get\_sampler\_params(std::vector< double > &values)

**Definition:** base\_nuts.hpp:171

stan::mcmc::nuts\_util::log\_u

double log\_u

**Definition:** base\_nuts.hpp:17

stan::math::min

double min(const double a, const double b)

**Definition:** min.hpp:7

stan::mcmc::base\_hmc::seed

void seed(const Eigen::VectorXd &q)

**Definition:** base\_hmc.hpp:51

stan::mcmc::base\_hmc::rand\_uniform\_

boost::uniform\_01< BaseRNG & > rand\_uniform\_

**Definition:** base\_hmc.hpp:140

stan::mcmc::base\_nuts::build\_tree

int build\_tree(int depth, Eigen::VectorXd &rho, ps\_point \*z\_init\_parent, ps\_point &z\_propose, nuts\_util &util)

**Definition:** base\_nuts.hpp:181

stan::agrad::log

fvar< T > log(const fvar< T > &x)

**Definition:** log.hpp:15

stan::agrad::exp

fvar< T > exp(const fvar< T > &x)

**Definition:** exp.hpp:16

stan::mcmc::base\_nuts::max\_delta\_

double max\_delta\_

**Definition:** base\_nuts.hpp:249

stan::mcmc::base\_nuts::write\_sampler\_params

void write\_sampler\_params(std::ostream &o)

**Definition:** base\_nuts.hpp:159

stan::mcmc::sample

**Definition:** sample.hpp:13

stan::mcmc::base\_nuts::set\_max\_delta

void set\_max\_delta(const double d)

**Definition:** base\_nuts.hpp:49

stan::common::sample

void sample(stan::mcmc::base\_mcmc \*sampler, int num\_warmup, int num\_samples, int num\_thin, int refresh, bool save, stan::io::mcmc\_writer< Model, SampleRecorder, DiagnosticRecorder, MessageRecorder > &writer, stan::mcmc::sample &init\_s, Model &model, RNG &base\_rng, const std::string &prefix, const std::string &suffix, std::ostream &o, StartTransitionCallback &callback)

**Definition:** sample.hpp:13

stan::mcmc::nuts\_util::n\_tree

int n\_tree

**Definition:** base\_nuts.hpp:22

stan::mcmc::nuts\_util::sum\_prob

double sum\_prob

**Definition:** base\_nuts.hpp:23

stan::mcmc::base\_hmc::integrator\_

I< H< M, BaseRNG >, P > integrator\_

**Definition:** base\_hmc.hpp:134

stan::mcmc::base\_hmc::epsilon\_

double epsilon\_

**Definition:** base\_hmc.hpp:143


---

[ Stan Home Page ]

*© 2011–2014,
Stan Development Team.*
